# Rapid microRNA detection method based on DNA strand displacement for ovarian cancer cells

**DOI:** 10.7150/jca.81050

**Published:** 2023-03-13

**Authors:** Gege Sun, Congzhou Chen, Xin Li, Shangwei Hong, Chuanqi Gu, Xiaolong Shi

**Affiliations:** 1 Department of Gynecology 2, Renmin Hospital of Wuhan University, 430072, Wuhan, China; 2 School of Computer Science, Beijing University of Technology, 100124, Beijing, China; 3 Institute of Computing Science & Technology, Guangzhou University, 510006, Guangzhou, China

**Keywords:** ovarian cancer, miR-21 detection, DNA isothermal strand displacement reaction

## Abstract

The current cancer detection methods are heavily dependent on the component analysis of corresponding cancer antigens. There is a lack of effective and simple clinical methods of ovarian cancer screening, which hinders the early identification for ovarian cancer and its treatment. To develop a simple and rapid method for quantitative analysis of ovarian cancer, we developed a DNA strand displacement-based method and finished the rapid detection of miR-21 in ovarian cancer cells within 5 min by a one-step isothermal reaction. The fluorescence intensity trajectory had a good linear relationship with miR-21 concentrations in the range of 100 fM-100 nM, with a lower limit of 6.05 pM. This detection method is simple, faster, and accurate. Besides, it can be applied to detect the miRNA biomarkers of other cancers by changing the preset sequences of toehold.

## Introduction

Ovarian cancer is the most aggressive gynecologic malignancy. According to the global cancer statistics, ovarian cancer-related deaths accounted for 23.3% of the total incidences in 2018 1, ranking among the top causes of cancer-related deaths. Ovarian cancer has an insidious onset and is difficult to diagnose, while the mortality rate in the middle and late stages is high. The lack of early identification methods makes it difficult for effective intervention and treatment 2.

High miR21 expression is regarded as a crucial indicator of ovarian cancer in clinical ovarian cancer examinations. miR21 is expected to have great potential in tumor diagnosis and therapy 3, 4. Dynamic monitoring of miR-21's expression in human circulating blood could provide intuitive and visual guidance for early tumor diagnosis, chemotherapy efficacy evaluation, and prognosis prediction. miR21's upregulation promotes the proliferation and invasion of ovarian cancer cells, inhibits apoptosis, and is strongly associated with the development of chemotherapy resistance 5.

Relevant literature has found that miR-21 can be used as a biomarker for the diagnosis of a variety of cancers. For example, some studies have shown that miR-21 in extracellular vesicles in cerebrospinal fluid can be used as a biomarker for the diagnosis of glioma. miR-21 is also significant in the diagnosis of prostate cancer, and miR-21 expression in serum and stool can be used as a potential diagnostic indicator for colorectal cancer. A related meta-analysis also showed that serum miR-21 has good diagnostic value in ovarian cancer 6, 7. Besides, higher expression of miR-21, 92 and 93 in the serum of patients with ovarian cancer before CA-125 elevation suggests that miR-21 can be used as a marker for early diagnosis 3. Except for the diagnostic value, miR-21 affects the proliferation and apoptosis of ovarian cancer cells by regulating the PTEN/PI3K/AKT pathway 4 and the jagged-1 pathway 8, and it regulates ovarian cancer cell invasion, migration, and colony formation through the Wnt/CD44v6 pathway 9. Upregulation of miR-21 was observed to be positively correlated with tumor grade and stage in ovarian cancer tumor tissues 10. Therefore, it is reasonable to consider miR-21 as a specific diagnostic marker for the detection of ovarian cancer.

Currently, miRNA biomarker-based ovarian cancer screening mainly relies on exponential amplification methods 11, 12, such as quantitative reverse transcription polymerase chain reaction (qRT-PCR) 13, Northern blotting 14, microarray 15, and RNA sequencing 16. However, a slight variation in the serum sample might have a great deviation on these ultra-high sensitivity techniques. Therefore, it is extremely demanding in terms of experimental technique. Meanwhile, studies have found that miR-21 exists in various cells, tissues, and blood samples 17. Ultrasensitive detection findings can only reveal the target gene's presence but not its quantity. For an early diagnosis, the expression should be quantified, and the concentration range for a disease diagnosis should be determined.

Therefore, it is meaningful to design a method that is convenient, fast, non-PCR dependent, and can quantify the miRNA expression in serum samples. To solve this problem, we designed a toehold-mediated strand displacement reactions (TSDR)-based method to quantify the expression of miRNA21 and to achieve the early diagnosis of ovarian cancer.

TSDR was first proposed by Yurke et al. 18. It involves three main processes as follows: toehold binding, branching chain migration, and chain dissociation 19. Specifically, TSDRS is driven by Gibbs free energy. A short invading strand binds to the toehold region on the DNA or RNA double strand, undergoes higher affinity base complementary pairing through progressive strand displacement, and then gradually replaces the original single strand bound to the underlying gating strand. Finally, the complex forms a more stable double-strand structure 20, 21. By changing the sequence and length of the toehold, the strand displacement rate constant may vary by more than 106 folds 22. This kinetic change is similar to the strand replacement reaction with DNA duplex and RNA duplex as substrates 23.

So far, TSDRs have been applied in various fields, including biocomputing systems 24-27, artificial neuro systems 28, 29, bio-electronical circuits 30, 31, and biosensors 32, 33. In the field of biosensors, many remarkable works have been accomplished.

Mohammadniaei et al. designed a nonenzymatic isothermal strand displacement and amplification assay (NISDA) based on TSDR. They used intercalating nucleic acid technology to enhance binding affinity. Severe acute respiratory syndrome coronavirus 2 (SARS-CoV-2) was detected by NISDA 34. Gao et al. 35 combined carbon dot (CD)-labeled fuel DNA and graphene oxide, and developed a fluorescent biosensor to detect miRNA let-7a. If let-7a is present, two consecutive toehold-mediated strand displacement reactions (TSDRs) are triggered, fuel DNA-CDs separate from the graphene oxide surface, and the fluorescence signal can be recovered. These novel biosensors consisted of two reactions, signal recognition and signal enhancement, which greatly improved the detection efficiency. Chen et al. 36 developed a new self-assembled DNA nanoprobe to measure the expression of miR-21. When miR-21 was present, three DNA hairpin probes equipped with free G-quadruplexes turned on assembly to form DNA nanospheres, which increased the fluorescence emission intensity of thioflavin.

Although these assays can detect target genes with high sensitivity, the operation is complicated and time-consuming, which is not conducive to the clinic. Besides, most of these assays only analyze a single indicator. Traditional assays detect the presence of target gene expression in a lesion as well as provide a qualitative judgment. The up- or downregulation of a target gene should be judged by an experienced physician. Due to these limitations, it is important and clinically relevant to develop a quantitative assay with convenient, fast, non-PCR properties.

Here, we propose a non-laboratory, enzyme-free, simple, and structurally stable isothermal strand displacement reaction for the rapid analysis of miR-21. We designed two toehold domains in the loop region and 3' end free stem region of the hairpin probe HP1. Generally, toehold either locates at the loop of the hairpin strand or at the stem of the hairpin. The reaction efficiency would be hampered when the toehold domain sites at the loop. As the toehold domain "hidden" in the metastable DNA structure 37. When the toehold domain sits at the stem of the hairpin, it will affect the efficiency of DSD reactions. Toehold overhang at the end of the hairpin stem region requires internal fluorophore or quencher labeling, which imposes more cost and improper quenching of the fluorophore. More importantly, these molecular tools are not efficient for long DNA or RNA targets (the whole genome), and they typically use enzymatic steps to produce short cDNAs for subsequent signal amplifications 34. The addition of the two toehold domains does not affect the labeling of the fluorophore and quencher but also facilitates the invasion of the target chain and accelerates the reaction efficiency.

## Materials and Methods

### Materials and reagents

The ovarian cancer cell line (SKOV3) and human normal ovarian epithelial cell line (IOSE80) were purchased from China Center for Type Culture Collection (Wuhan, China). Fetal bovine serum (FBS) and Roswell Park Memorial Institute (RPMI) 1640 media were obtained from Gibco (Shanghai, China). Total RNA extraction kit, microRNA Reverse Transcription Kit, and 2× SYBR Green qPCR master mix were supplied by Sangon Biotech Co., Ltd. (Shanghai, China). RNA-related operations required the involvement of Rnase-free water.

### Preparation of the testing system

The sequence of miR-21 was obtained from the NCBI (National Center for Biotechnology Information). Hairpin DNA structures HP1 and HP2 were designed and free Gibbs energy (ΔG) calculations were performed with a Nupack webserver (http://www.nupack.org/), with the following parameters: T_m_ ≥ 37°C; ΔG ≤ -13.9 kcal mol^-1^; 30% ≤ GC% ≤ 55% (Table 1). Mimic-miR21, HP1, and HP2 in were solubilized with 1× TAE Mg^2+^ (40 mM Tris, pH = 8.2, 2 mM EDTA,12.5 mM Mg^2+^) buffer. Annealing of HP1 and HP2 was completed by gradual cooling from 95°C to room temperature in 5 min, and finally stored at 4°C. All oligonucleotide sequences are listed in Table 2.

### PAGE analysis

The 12% PAGE (polyacrylamice gel electrophoresis) was performed in 6 mL of 1× TAE buffer, 3 mL of polyacrylamide, 60 µL of 10% ammonium persulfate and 6 µL of tetramethylethylenediamine (TMED). Gels were set by leaving at RT for 20 min. The reacted DNA samples and 6× DNA loading buffer were mixed at a 5:1 ratio and the mixture was then added to the samples in a certain order. Electrophoresis was performed in 1× TAE running buffer at 80 V constant voltage for 120 min. Then, the products were placed in GelRed DNA gel stain solution for 15 min and the gel image was obtained using the JY04S-3C Gel Documentation Imaging System (Beijing).

### Serum stability analysis

HP1 and HP2 were incubated with 10% FBS in a metal bath at 37°C for 0-12 h. The difference in stability of HP1 and HP2 over time was confirmed via agarose gel electrophoresis.

### Cell culture and total RNA extraction

IOSE80 and SKOV3 cells were grown in RPMI 1640 with 1% penicillin-streptomycin solution and 10% FBS. The incubator was kept at a constant temperature of 37°C and filled with 5% CO2.

All tips and tubes used for RNA experiments were enzyme-free. Total RNA was extracted from the cells using the Trizol method. Add Trizol (1 ml) to a 6 cm dish, let stand for 2 min, then transfer to a new 1.5 ml tube. leave for 5 min at RT to achieve cell lysis. Add 200 μL of chloroform to each tube, mix upside down for 15 s, and allowed to stand for 2 min, and the tubes were centrifuged at 4°C and 12000×g for 15 min. RNA was then transferred to a new 1.5 mL tube. Next, 500 µL of isopropanol was added to each tube, mixed for 15 s, and then left to stand at RT for 5 min before centrifuging the samples at 4°C, 12,000 x g for 10 min for RNA precipitation. The upper supernatant was discarded and left to dry for 5 minutes. One mililiter of 75% ethanol prepared with DEPC water was added to each tube and centrifuged at 7500×g for 5 min at 4°C. To remove excess ethanol, discard the supernatant and air dry again for 10 minutes. Finally, add 30 μL of DEPC water to each tube to dissolve it.

### qRT-PCR and linear PCR

The mixture for microRNA reverse transcription contains 2 x miRNA qRT solution mixture (10 µL), miRNA qRT enzyme mixture (2 µL) and total RNA (2 µg), and made up with RNase-free water to 20 µL. The procedure was performed as follows: 37°C for 1 h and 85°C for 5 min. Then, the reaction products were placed in -20°C refrigerator for freezing and storage.

The newly synthesized cDNA were served as a template for qRT-PCR. The solution mixture was prepared as follows: 2× miRNA qPCR master mix (10 µL), forward primer(0.5ul) and reverse primer (0.5 µL), cDNA (2 µL), and RNase-free water (7 µL). The qPCR was run for 39 cycles as follows: 95°C for 5 s and 60°C for 30 s. The relative expression of cell samples was assessed with U6 gene expression as a reference (Table 2).

Linear PCR was performed using MicroRNA qPCR Kit. The reaction mixture was prepared as follows: 2× miRNA qPCR master mix (10 µL), miR21-forward primer (0.5 µL), cDNA (1 µL), and RNase-free water (8.5 µL). qPCR was run for 40 cycles as follows: 95°C for 5 s, 60°C for 10 s.

### miR-21 detection

The detection mixture consisted of 1 µL of HP1 (150±2ng/µL), 2 µL of HP2 (215±3ng/µL), 1 µL of miR-21 (80±2ng/µL) and 6 µL of RNase-free water. Incubate at 37°C for 60 min.

### Statistical analysis

Statistical analysis was carried out using GraphPad Prism 8. At least three independent replications were performed for each experimental data acquisition. Data were expressed as the mean ± standard deviation. An unpaired two-tailed t-test was used to calculate the difference between the two groups, and a p-value of < 0.05 was considered significant.

## Results

### Principle of assay

**Figure 1:**
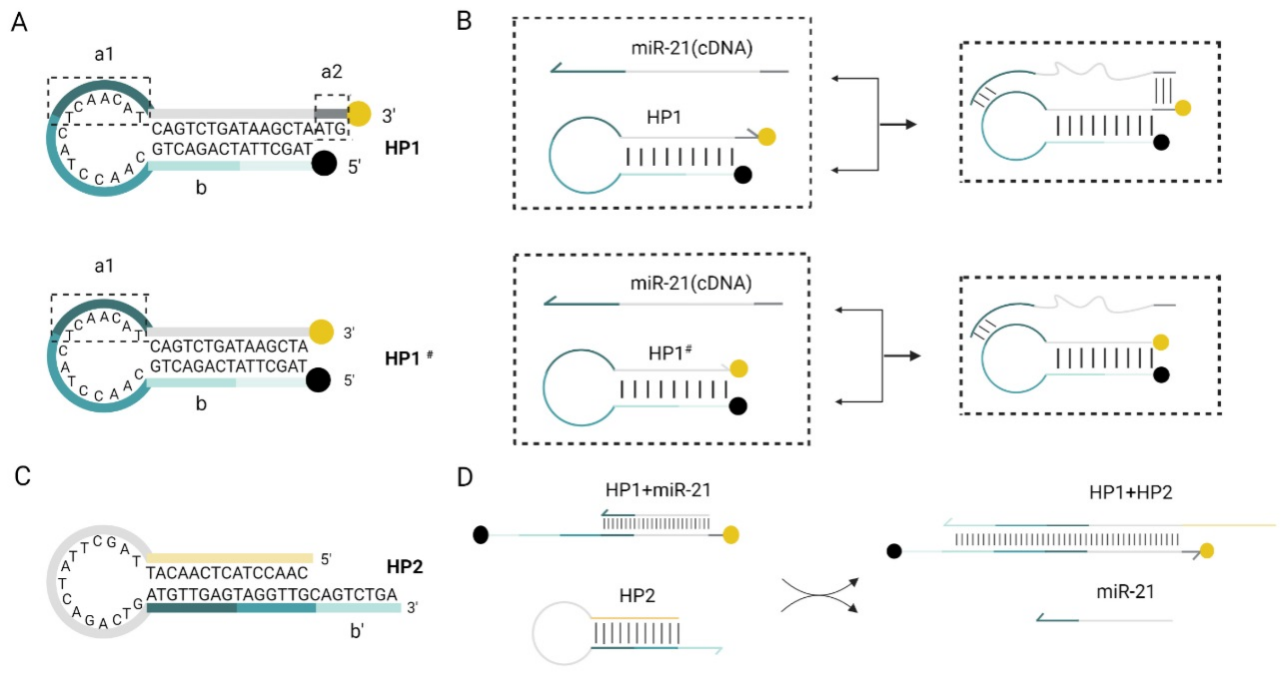
** Schematic diagram of the RNA detection system.** (A) Double toehold hairpin probe HP1 comparing with single toehold hairpin probe HP1^#^, a1 is the toehold domain in the loop region while a2 is a free toehold at 3' end. Black point at 5' end of HP1 indicates the BHQ1 quencher, and yellow point at 3' end indicates the 6-Fam fluorescent group. (B)Practical efficiency of the double toehold sequence. With the presence of miR-21 (cDNA), it can also play a dual role in binding to a2, which is more efficient than HP1^#^ application. (C) Schematic design of the hairpin probe HP2. (D) The reaction of miR-21 with HP1 to form an unstable double chain, and its chain substitution reaction with HP2 is shown in the flow chart.

**Figure 2:**
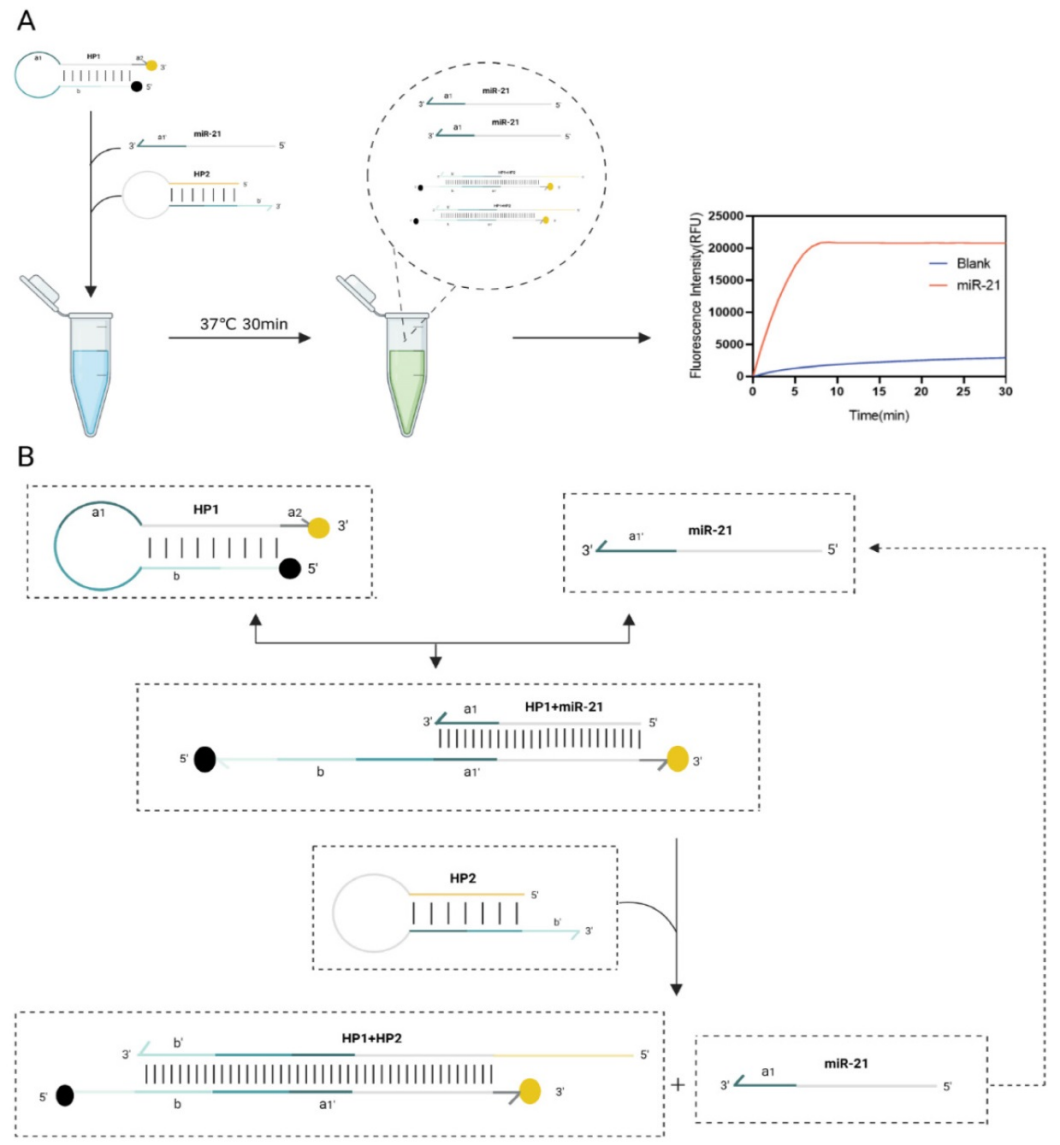
** Flow chart for rapid detection of miR-21**. (A) Scheme diagram of the testing program. (B) The reaction mixture includes 3 critical components, hairpin probes HP1, HP2 and miR21. 2-step toehold-mediated strand replacement reaction occurs sequentially in the presence of invading strand RNA/DNA, and fluorescence detection is performed at 37°C, 30 min.

As shown in Fig. 1 and Fig.2, this RNA detection method contains only two DNA hairpins, HP1 and HP2. Mimic-miR21 (Synthesized ss-DNA with the same sequence of miR21) was used as the invasion chain to validate the feasibility of the protocol. BHQ1 quencher and FAM fluorophore were labeled at the 5' end and 3' end of HP1, respectively.

When miR-21 was present, it recognized double toehold a1and a2 of HP1. HP1 was gradually unchained and the fluorescence is restored. After this DNA strand displacement reaction, a new toehold domain b of HP1 was exposed. It could pair with the b' domain of HP2, free outside the stem-loop, which in turn gradually displaces miR-21 a more stable HP1:HP2 double chain, and the fluorescence can be continuously excited. Finally, the displaced miR-21 continued into the next DNA strand displacement reaction cycle. This fluorescence amplification process can detect RNA with high sensitivity. After 30 min of reaction at 37°C, almost all HP1 and HP2 were bound into stable duplexes and the fluorescence signal intensity increased significantly.

### Design of molecular structure

We selected mir-21 to be tested in our study. Due to the stringent requirements for temperature and enzyme-free environment for conducting RNA-related experiments, previously published conditions 18 were not conducive to demonstrating the superiority of our assay. Therefore, we chose mimic-miR21 for the follow-up experiments.

**Figure 3:**
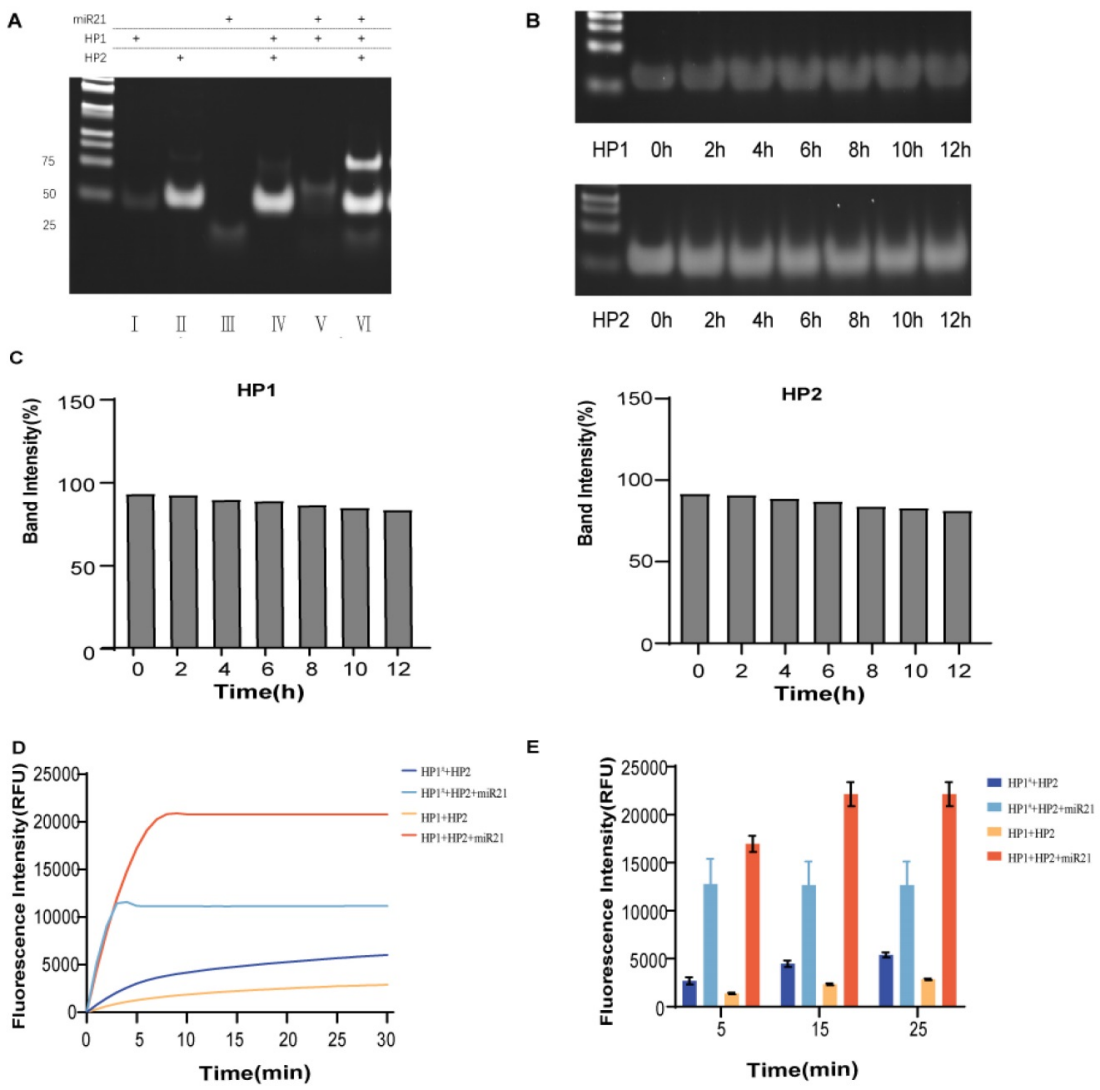
** Rapid miR21 detection by the double toehold hairpin system**. (A) PAGE analysis of the feasibility of this assay protocol. Lane 1: HP1. lane 2: HP2; lane 3: mimic-miR21; lane 4: HP1+HP2; lane 5: HP1+mimic-miR21; lane 6: for HP1+HP2+mimic-miR21. (B) PAGE analysis of the stability of hairpin probes HP1 and HP2 at 12 h incubation in 10% FBS. (C) Semiquantitative analysis of the relative band intensity. (D) Fluorescence was collected by real-time quantitative PCR instrument to compare the difference in the change of reaction rate between the double toehold HP1 and single toehold HP1^#^ participation. (E) The fluorescence values of each group at 5 min, 15 min and 25 min were taken as graphs to quantitatively compare the difference in the rate of change of the reaction involving a double toehold HP1 and a single toehold HP1^#^. Error bars were calculated in three independent experiments.

Validation of the advantages of the dual toehold was also performed. Two toehold sequences, a1 and a2, were designed on HP1. The toehold a1 was located in the loop region of HP1, which could bind to miR-21 and undergo a strand displacement reaction. The toehold a2 was an ATG sequence free outside the stem at the 3' end of HP1. HP1 with only toehold a1 and the HP1 with double toehold a1 and a2 were incubated with HP2 and miR-21, respectively, at 37°C. Comparing the reaction time and fluorescence signal intensity between the two groups, we found that the addition of toehold a2 did not affect the reaction rate (Fig. 3D and E). The double toehold sequence was designed to apply the assay in practice better. We first amplified the cDNA generated via reverse transcription with the tailing method, and the amplified product base extended out of the CAT sequence, which had complementary base pairing with the toehold a2. The unzipping of the hairpin structure of HP1 was because the toehold sequence free outside the hairpin structure was more likely to bind to the foreign single strand than the toehold sequence contained in the loop region. Therefore, the addition of toehold c greatly increased the efficiency of DNA strand displacement in practical applications. Meanwhile, the RNA single strand could bind directly to the toehold a1. The addition of toehold a2 did not affect the invasion of RNA.

### Optimization of optimal conditions

**Figure 4:**
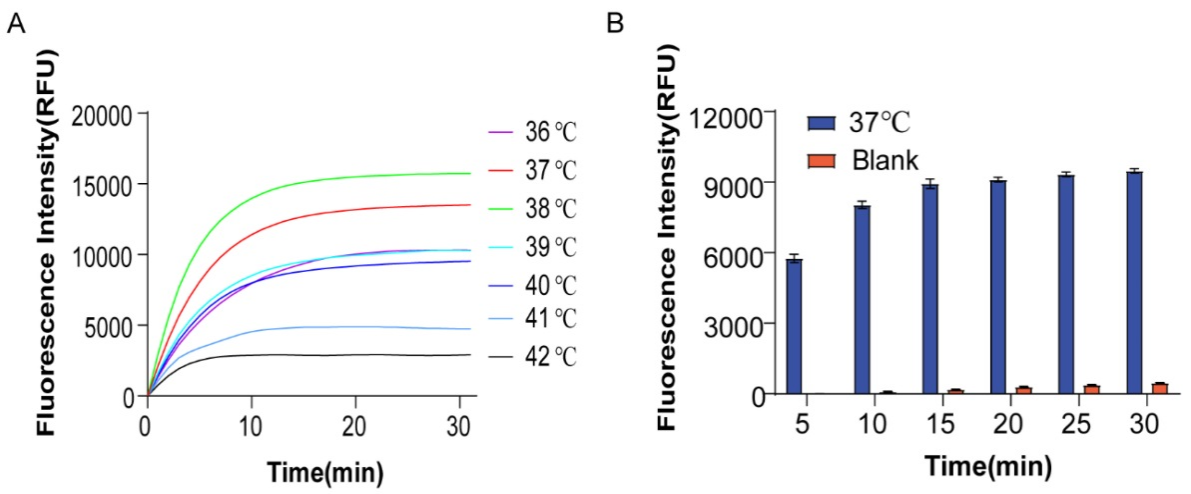
** Optimization of reaction conditions.** (A) Optimal reaction temperature for this assay method. (B) Optimal reaction time for this assay method. Error bars were calculated in three independent experiments.

The stability of HP1 and HP2 was affected by temperature. At incubation temperatures lower than the optimal temperature, the free energy of the hairpin decreased and its stability increased, making it less likely to be unzipped by the invasion chain. When the incubation temperature was too high, the HP1 and HP2 would spontaneously unwind to form a small amount of HP1:HP2 duplexes in the presence of no target molecules, producing a certain fluorescence leakage and giving a false positive result. Therefore, we optimized the incubation temperature by comparing the fluorescence intensity at different incubation temperatures. Fig. 4A shows the fluorescence intensity change curve of the input group incubated at different temperatures of 36 - 42°C at 30 min. When incubated at 36-38°C, the fluorescence intensity was found to be proportional to temperature. After about 5 min, one could make a clear diagnosis by the obvious difference in fluorescence response, and the fluorescence intensity reached the maximum within 15 min. At reaction temperatures above 38°C, the fluorescence intensity gradually decreased with increasing incubation temperature. At 38°C, the fluorescent group and the quencher were stably separated and the fluorescence signal intensity was the strongest due to the stable double strands formed by HP1 and HP2. When the temperature exceeded the optimum, the duplex became unstable, in which case the partially free single-strand HP1 became aggregated. The fluorophore and quencher were in close proximity to each other, resulting in a decrease in fluorescence signal intensity, resulting in a decrease of fluorescence signal intensity. signal intensity. Although the fluorescence intensity reached its maximum at 38°C, similar experimental results were obtained for the reaction at 37°C. To explore the clinical value better, 37°C was chosen as the optimum temperature for subsequent experiments.

We also optimized the optimal reaction time. As shown in Fig. 4B, after adding miR-21, the fluorescence signal was not recovered after incubation at 37°C for 15 min, implying that the reaction was completed. Therefore, we selected 15 min for the optimum reaction time. At the same time, it was easy to observe the results as the obvious fluorescence effect appeared after 5 min incubation at 37°C, indicating fast detection.

### Validation of sensitivity and specificity

**Figure 5:**
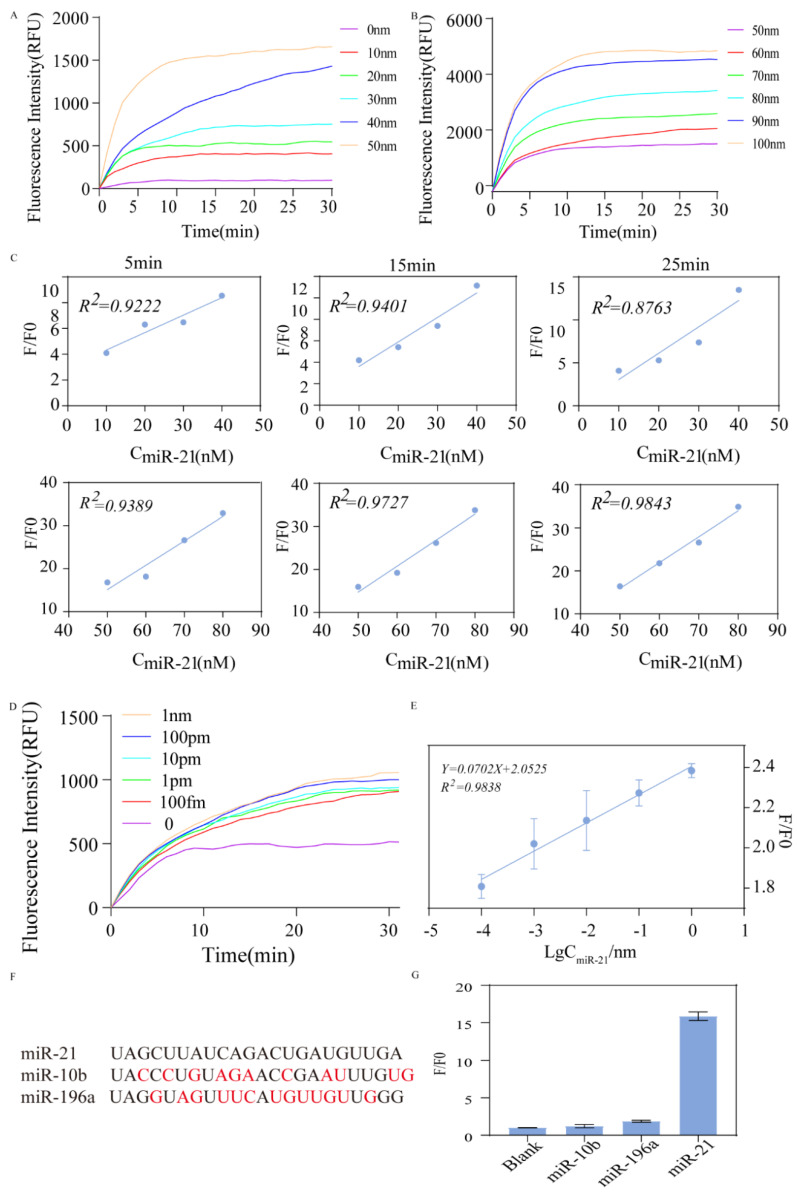
** Validation of sensitivity and specificity**. (A) Fluorescence change when miR-21 concentration is at 0-50nm. (B) Fluorescence change when miR-21 concentration was at 50-100 nm. (C) Changes in F/F0 with miR-21 concentration when the reaction time was 5 min, 15 min, 25 min, respectively. (D) Fluorescence change when miR-21 concentration was varied at 1nm, 100pm, 10pm, 1pm, 100fm. (E) Change of F/F0 with miR-21 concentration. (F) The sequences of the detection of different miRNA targets. (G) Selectivity of the assay for miRNA-21 detection.

The sensitivity of the assay was assessed by testing it under optimal reaction conditions. Fig. 5A and 5B show that the fluorescence intensity increased with the increase of target chain concentration (10-100 nm). Fig. 5C shows a good linear relationship between the ratio of fluorescence signal intensity/blank control fluorescence signal intensity (F/F0) and C_miR-21_ for each group after 5, 15, and 25 min.

To further validate the detection sensitivity limit of this protocol, we observed changes in fluorescence signal intensity when miR21 concentrations were 10^-4^, 10^-3^, 10^-2^, 10^-1^, and 1 nm. As shown in Figure 5D, the higher the concentration of miR21, the faster the fluorescence signal was saturated. Even at very low template concentrations (down to 100 fm), a rapid fluorescence effect was detected within 30 min. The corresponding F/F0 was graphed against LogCmiR-21 as shown in Fig. 5E to obtain a calibration curve with a linear relationship. The regression equation is Y=0.0702X+2.0525 (R2=0.9838) (Y means F/F0 and X means the concentration of miR-21). The LOD was 6.05 pm.

To assess the specificity of the method, we added different invasion chains under the same experimental conditions. The fluorescence signal intensity of miR-21, miR-10b, miR-196a, and blank control was compared. The corresponding F/F0 for each group was 1, 1.22, 1.87, and 15.87, respectively. A strong fluorescent signal was observed with miR-21 as the input, while the nonspecific molecule's fluorescence signal intensity approached that of the blank, showing the extremely high specificity of the scheme. There was little interference or crosstalk between non-target chains (Fig. 5F and G).

### Detection of miR-21 in actual samples

**Figure 6:**
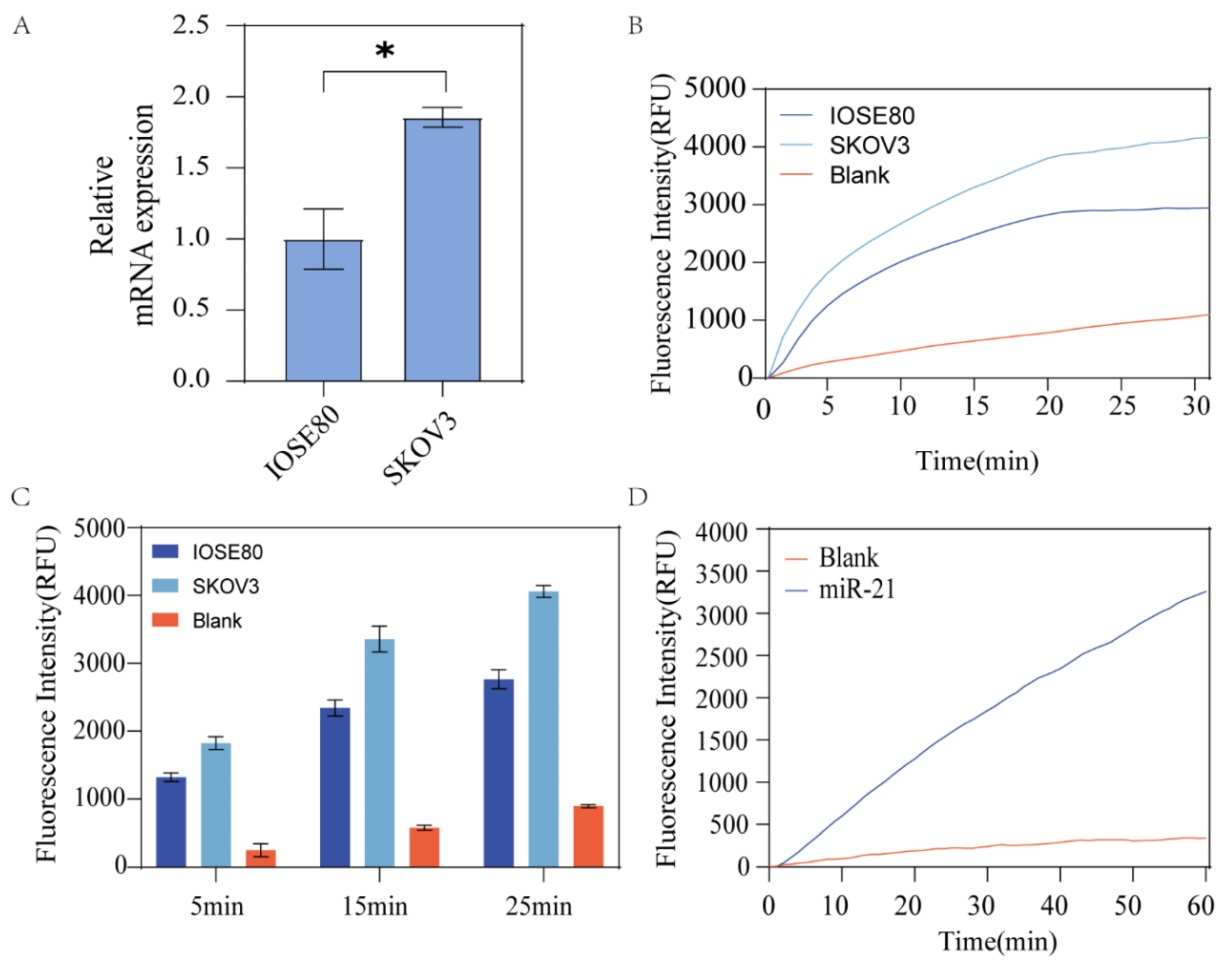
** Rapid Detection of ovarian cancer cells.** (A) qRT-PCR detection of miR-21 expression in IOSE80 and SKOV3. (B) After extracting total RNA from IOSE80 and SKOV3, respectively, the reverse transcribed product was used as the invasion strand to observe the fluorescence changes. (C) The fluorescence changes of different groups at different incubation times of 5 min, 15 min and 25 min were quantified by doing histogram analysis. (D) Fluorescence change over time by using the synthesized miR-21 directly as input.

As Fig. 6A shown, RNA was extracted from SKOV3 and IOSE80, and cDNA was obtained via reverse transcription to verify the actual application of the assay. First, the expression of miR21 in two cell lines was verified using qRT-PCR, and the results revealed that miR21 was overexpressed in SKOV3 (Fig. 6B). cDNA was amplified and the products were mixed with HP1 and HP2 after 40 cycles and incubated at constant temperature for 15 min. The results showed different expression levels of miR21 in the two types of cell lines, and the differences with the blank control group were obvious (Fig. 6C). This result was in accordance with the RT-qPCR assay. Similarly, we used synthetic miR-21 RNA samples as input to verify the feasibility of this scheme and found that the RNA as the invasion strand could also catalyze the strand replacement reaction with satisfactory experimental results (Fig. 6D).

These results showed that miR-21 expression was upregulated in SKOV3, and the results were statistically different. Figure 6B shows the results of the actual sample assay in the reaction, showing that the SKOV3 group produced stronger fluorescence compared to the IOSE80 group, and the assay results converged with qrt-pcr, validating the feasibility of the assay protocol.

## Conclusion

In this study, we developed a rapid isothermal signal amplification assay without enzyme involvement for the detection of miR-21 in ovarian cancer cells. We added target genes, HP1 and HP2 to amber EP tubes, and the fluorescence signal can be detected. The lower detection limit of LOD is 6.05 pM. In addition, quantitative analysis based on the change in fluorescence intensity at miR21 concentrations from 10-100 nM revealed a good linear relationship between miR21 concentration and F/F0. The serum stability results of HP1 and HP2 showed superior stability of the molecular structures.

To apply this assay, we built the hairpin probe HP1 with double toehold regions. Compared with single toehold assays, such as NISDA, adding a short sequence of 3 base length footholds at the free end does not affect fluorophore termination. Additionally, this strategy can make HP1 more susceptible to hybridization with miR-21, which forms an irreversible and stable double-stranded structure. The twin-toehold design is RNA and DNA compatible. This reduces response time and boosts fluorescence intensity.

Our study revealed that this assay can rapidly detect mir21 in ovarian cancer cells within 5 min at the optimal temperature of 37°C, with a limit of detection as low as 6.05 pM. Meanwhile, the quantitative analysis revealed a good linear relationship between F/F0 and the concentration of miR-21 in the range of 10 nm-100 nm. Further, the assay was able to distinguish between miR-21 expression in ovarian cancer cells and normal ovarian epithelial cells. The detection results converged with the validation of qPCR with high specificity. It is well known that there is still a lack of clinical methods to perform large-scale screening of people at high risk for ovarian cancer development, and our assay is simple, rapid, and combines high sensitivity and specificity for all types of medical sites. By changing the sequence of the toehold, we are able to build a universal platform for the detection of other genes.

## Figures and Tables

**Table 1:** Analytical data corresponding to the predicted binding energy (ΔG) at 37 °C, melting temperature (Tm), GC content (GC%), and base pair formation of different probes

Target gene	Oligo	37°C ΔG (kcal mole^-1^)	*T*m (°C)	GC %	Base pairs
miR21	HP1	-13.9	64,1	39.6	15
	HP2	-15.27	66.5	41.5	15
	HP1:HP2 duplex	-53.46	-	-	30
	HP1:miR-21 (cDNA) duplex	-30.44	-	-	25
	HP1:miR-21 duplex	-27.67	-	-	22

**Table 2:** Oligonucleotides sequences

Oligo name	Sequence 5'-3'
miR-21	UAGCUUAUCAGACUGAUGUUGA
Mimic-miR21	TAGCTTATCAGACTGATGTTGA
HP1-FAM/BHQ	BHQ1-TAGCTTATCAGACTGCAACCTACTCAACATCAGTCTGATAAGCTAATG-FAM
HP1^#^-FAM/BHQ	BHQ1-TAGCTTATCAGACTGCAACCTACTCAACATCAGTCTGATAAGCTA-FAM
HP2	CAACCTACTCAACATTAGCTTATCAGACTGATGTTGAGTAGGTTGCAGTCTGA
miR-10b	UACCCUGUAGAACCGAAUUUGUG
miR-196a	UAGGUAGUUUCAUGUUGUUGGG
miR-21 forward primer	TTTTTTTTTTCAACAT
miR-21 reverse primer	AGTGCAGGGTCCGAGGTATT
U6 forward primer	AGAGAAGATTAGCATGGCCCCTG
U6 reverse primer	ATCCAGTGCAGGGTCCGAGG
